# Protection of Historical Wood against Microbial Degradation—Selection and Application of Microbiocides

**DOI:** 10.3390/ijms17081364

**Published:** 2016-08-22

**Authors:** Anna Koziróg, Katarzyna Rajkowska, Anna Otlewska, Małgorzata Piotrowska, Alina Kunicka-Styczyńska, Bogumił Brycki, Paulina Nowicka-Krawczyk, Marta Kościelniak, Beata Gutarowska

**Affiliations:** 1 Institute of Fermentation Technology and Microbiology, Lodz University of Technology, 90-924 Łódź, Poland; katarzyna.rajkowska@p.lodz.pl (K.R.); anna.otlewska@p.lodz.pl (A.O.); malgorzata.piotrowska@p.lodz.pl (M.P.); alina.kunicka@p.lodz.pl (A.K.-S.); beata.gutarowska@p.lodz.pl (B.G.); 2 Laboratory of Microbiocide Chemistry, Faculty of Chemistry, Adam Mickiewicz University, 60-780 Poznań, Poland; brycki@amu.edu.pl; 3 Department of Algology and Mycology, University of Lodz, 90-237 Lódź, Poland; paulina_nowicka84@interia.pl; 4 Auschwitz-Birkenau State Museum, 32-603 Oświęcim, Poland; marta.koscielniak@auschwitz.org

**Keywords:** historical wood protection, microbiocides, quaternary ammonium compounds, disinfection

## Abstract

The aim of this study was to select effective and safe microbiocides for the disinfection and protection of historical wooden surfaces at the former Auschwitz II-Birkenau concentration and extermination camp. We tested seven active compounds against bacteria and moulds, of which didecyldimethylammonium chloride and *N*-(3-aminopropyl)-*N*-dodecylpropane-1,3-diamine were effective even at 0.02%–2%. Subsequently, eight microbiocides containing the selected active ingredients were chosen and applied three times on the surface of wood samples colonized by bacteria and moulds. ABM-1 and ABM-2—6% solution; Rocima 101—8%; Preventol R 80—12%; Acticide 706 LV—15% and Boramon—30% were the most effective disinfectants. Under laboratory conditions, ABM-1, Boramon and Rocima 101 ensured antimicrobial protection of new wood samples for six months. In situ, 30% Boramon and 8% Rocima 101 applied by spraying effectively protected the historical wood from bacterial and mould growth for 12 and 3 months, respectively. Colour and luminance of the new wood were not altered after exposure to the biocides. Boramon and Rocima 101, applied by the spraying method, caused no significant change in the colour of the historical wood. Results from this study were used to develop a procedure for the protection of wood in historical buildings against biodeterioration.

## 1. Introduction

For centuries, wood has been used in the construction of houses, ships, weapons and various tools, among other things. Due to its chemical composition, this natural organic material is easily colonized by microorganisms and insects, leading to its deterioration under conditions favourable for their growth [1,2,3,4]. 

Wood damage by bacteria and fungi involves degradation of cellulose, hemicellulose and lignin. This leads to aesthetic deterioration of the surface (peeling, delamination, discoloration) and, above all, structural and mechanical changes (reduced strength, hardness) [1,5,6,7]. However, properly maintained and treated wood can last for several years. 

Different chemical compounds have been regularly used, for about 200 years, in order to protect wood from damaging factors [8]. Initially, fluorine, arsenic, chromium and copper compounds were utilized, which were often toxic to humans. Chemicals used to protect wood were first mentioned in ancient times, e.g., precious wood soaked in cedar oil by Egyptians [2,9]. Currently, the most commonly used preservatives are copper compounds, chromium, boric acid, azoles or quaternary ammonium compounds [10,11]. Research is also being conducted on ionic liquids and fatty acids, which can be used in the protection of timber [12,13,14]. 

Chemical wood preservatives can be divided into impregnates and preservatives, which are used to protect wood against biological agents and fire, and biocides, which are applied to remove and neutralize biological agents already present in the wood [2,15]. Biocides include water-borne, oil-borne and organic solvent-based substances. Water-borne preservatives have been widely used in recent years [11]. However, these chemical preservatives have an impact on the environment and humans. The use of biocides for wood preservation must be authorized in accordance with the Biocidal Regulation 528/2012 [16].

Currently, preservation of wood (especially historical wood) is often a complex and multistage process. The following factors must be taken into account: structural (type of wood, its durability and storage conditions); biological; and chemical (previous impregnation, preservatives, etc.). Furthermore, both environmental and economic aspects must be considered when using biocides. In the case of historical wood, the impact of chemicals on the material is extremely important, as it can lead to its discoloration [2,17]. Improper use of preservatives may result in irreversible damage of historical objects that are often an invaluable heritage of past centuries.

The aim of this study was to select safe and effective microbiocides, for disinfection and protection of historical wooden surfaces at the former Auschwitz II-Birkenau concentration and extermination camp; this camp was part of the Auschwitz-Birkenau in Oświęcim, Poland. The scope of the work included:

- Selection of active biocidal substances against the microflora isolated from historical wood at the former Auschwitz II-Birkenau concentration and extermination camp;
- Selection of biocides containing the selected active compounds;
- Checking the activity and assessing the effectiveness of the biocides against microbes colonizing the wood surface;
- Protecting the wood samples from further contamination with microorganisms, under laboratory and in situ conditions;
- Evaluating the impact of biocides on new and historical wood colour and luminance;
- Developing a procedure for the protection of historical buildings from biodeterioration.

## 2. Results

### 2.1. Active Compounds

The quantitative and qualitative analysis of microorganisms colonizing wooden surfaces, such as doors, floors, bunk beds, door frames, beams and structural walls in the barracks at the former Auschwitz II-Birkenau concentration and extermination camp, was carried out in a previous study [18]. The results enabled us to determine the extent of contamination, and to select the dominant species of microorganisms. This was followed by inhibiting the microbial growth through the use of microbiocides. We evaluated the sensitivity of microorganisms to seven compounds commonly used as active ingredients in disinfectants: didecyldimethylammonium chloride (DDAC), *N*-(3-aminopropyl)-*N*-dodecylpropane-1,3-diamine (APDA), hydrogen peroxide (HP), glutaraldehyde (GA), sodium hypochlorite (SH), boric acid (BA), and lactic acid (LA). The susceptibility of bacteria to five of the seven compounds tested was similar (Table 1). The only exception was boric acid, for which the minimal inhibitory concentration (MIC) ranged from 2% to 8%, and was different for each of the tested strains. DDAC and APDA showed maximum bactericidal activity, with an MIC of 0.02%. Only *Pseudomonas fluorescens* showed low sensitivity to DDAC (MIC 7%).

*Engyodontium album* was considered the most sensitive of all moulds tested (Table 1). DDAC and APDA had the lowest MICs and maximum antimicrobial activity.

The compounds evaluated are ranked by their decreasing antimicrobial activity: *N*-(3-aminopropyl)-*N*-dodecylpropane-1,3-diamine (APDA) > didecyldimethylammonium chloride (DDAC) > sodium hypochlorite > hydrogen peroxide > glutaraldehyde > boric acid > lactic acid. Based on these results, two active ingredients were selected for further study: didecyldimethylammonium chloride, belonging to quaternary ammonium salts, and *N*-(3-aminopropyl)-*N*-dodecylpropane-1,3-diamine, a polyamine. These compounds are active ingredients in a variety of formulations available on the market.

### 2.2. Biocides

Based on data from the Office for Registration of Medicinal Products, Medical Devices and Biocidal Products (List of Biocides, Part I, including information about products, which have been granted a marketing authorization [19]), six biocides were selected for testing, and two preparations (ABM-1 and ABM-2) were prepared specifically for the project: “Research on the biological corrosion of objects in the Auschwitz-Birkenau State Museum in terms of identification and control of biological agents, Phase II. A study on the selection of chemicals for the control of microorganisms and algae, and for wood and mineral surface protection against their development”. In order to determine the spectrum of activity of the compounds, sensitivity of mixed cultures of microorganisms was evaluated to the selected biocides. Since the generation times and resultant growth in the test environment differed for bacteria and moulds, the assays were performed separately for each group (Table 2). Mixed strains of bacteria showed higher sensitivity than moulds. All biocides are based on quaternary ammonium compounds (see Section 4.3. Materials and Methods). Acticide LV 706 (10%) was the only biocide that inhibited growth of both bacteria and moulds. The results allowed the determination of optimal formulation concentrations (Table 2), which are effective in growth inhibition of microorganisms.

### 2.3. The Activity of Biocides against Microorganisms Colonizing the Wood Surface

**Figure 1:**
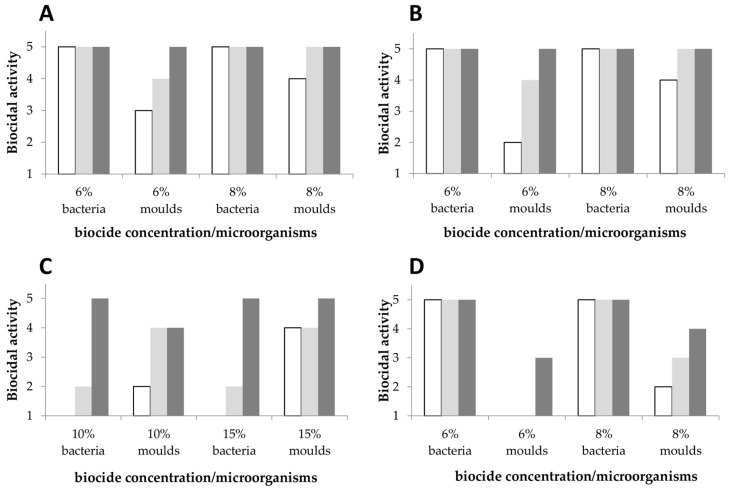

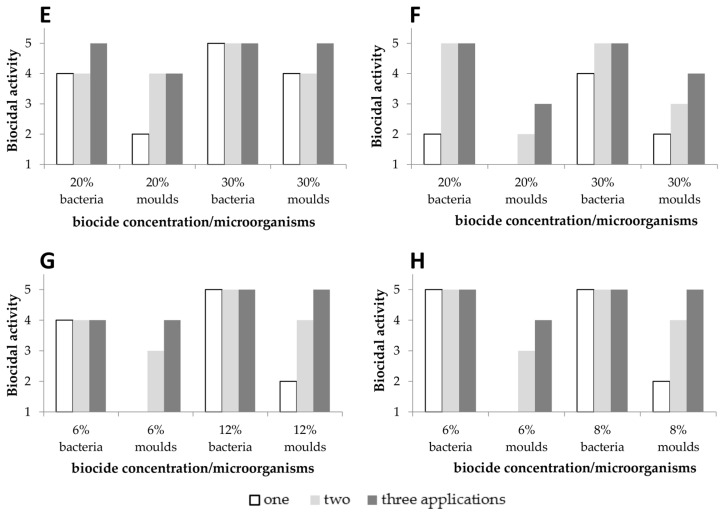
Effectiveness of disinfectants: ABM-1 (**A**); ABM-2 (**B**); Acticide LV 706 (**C**); Atoxyn (**D**); Boramon (**E**); Mycetox B’ (**F**); Preventol R80 (**G**); and Rocima 101 (**H**) against mixed populations of bacteria and moulds on wood: one (white bar graph), two (grey bar graph) and three (dark grey bar graph) applications.

In addition to concentration, the number of biocide applications is a very important factor in the disinfection process. In the present study, each disinfectant was sprayed onto the surface three times at two different concentrations. In order to determine the activity of the biocides, a five-level scale was used (see Section 4.8. Materials and Methods). ABM-1, ABM-2, Atoxyn and Rocima 101 exhibited the highest antibacterial activity when applied to the wood (Figure 1). These substances showed antibacterial activity at a concentration of 6% (i.e., lower of the two studied concentrations) after a single spray. According to the scale adopted in this study (see Section 4.8. Materials and Methods), the number of bacteria reduced by 8 log units, indicating the efficiency of the formulation at 5 units of biocidal activity. To achieve the same effect, Mycetox B’ had to be applied twice at a concentration of 20%, while Boramon and Acticide LV 706 were effective after three applications at concentrations of 20% and 10%, respectively. Preventol R80 showed antimicrobial activity only at the higher concentration (12%) after a single application. Moulds colonizing the wood samples were less sensitive to the applied disinfectants (Figure 1). ABM-1 and ABM-2 were the most effective antifungal biocides; moulds were eliminated from the surface of the material after three applications at the lower concentration (6%), and two applications of 8%. Antifungal activity was also exhibited by Acticide LV 706, Boramon, Preventol R80 and Rocima 101, after three applications at the higher of the tested concentrations (15%, 30%, 12% and 8%, respectively). The other disinfectants did not show expected antifungal activity (biocidal activity < 4). Although the growth of moulds was reduced, it was not eliminated.

ABM-1 and ABM-2 at a concentration of 6%; Rocima 101 at 8%; Preventol R80 at 12%; Acticide LV 706 at 15% and Boramon at 30% were the most effective against mixed populations of bacteria and moulds on the wood. Each of these disinfectants must be applied three times for effective disinfection.

In addition to biocidal activity, the economic calculation was also considered i.e., the average cost of disinfectant consumption per 100 m^2^ of surface in a single spraying procedure. Preventol R80 was the most expensive (€22.8 for a solution at a concentration of 6%). ABM-1 and ABM-2 were the cheapest, not exceeding €2.5, which is nine times less than the cost of other disinfectants.

### 2.4. Protection of Wood Samples from the Development of Microorganisms: Laboratory Tests

Next, the biocides were evaluated for their effectiveness in wood preservation, against mixed cultures of bacteria and moulds. The results obtained under model conditions show that Rocima 101 and ABM-1 at concentrations of 8% applied by the fogging method, as well as Boramon at 30% and Rocima 101 at 8% applied by the spraying method, are highly effective in protecting non-historical wood against bacteria and moulds. No growth of microorganisms was recorded on the samples protected with these biocides, after six months of incubation. Differences between methods of biocides application are described in Section 4.6. Materials and Methods.

### 2.5. Protection of Historical Wood Samples from the Development of Microorganisms—In Situ Tests

**Figure 2:**
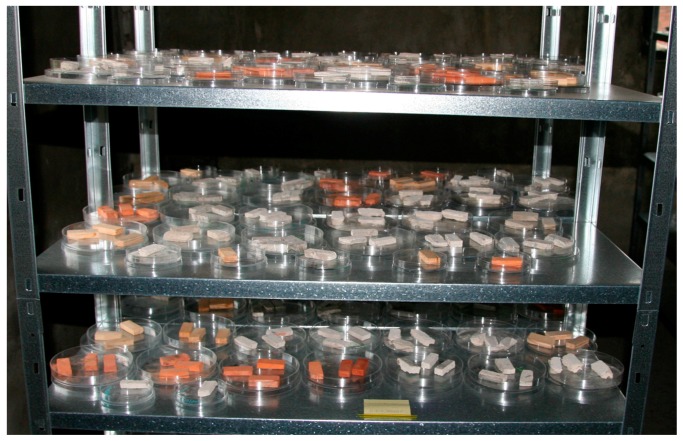
Historical wood samples after application of disinfectants, exposed in a barrack at the former Auschwitz II-Birkenau concentration and extermination camp.

In order to verify the laboratory tests, the same disinfectants were applied under in situ conditions. They were checked for effectiveness in preserving historical wood; the treated wood was then exposed in one of the barracks at the former Auschwitz II-Birkenau concentration and extermination camp, for 12 months (Figure 2).

**Figure 3:**
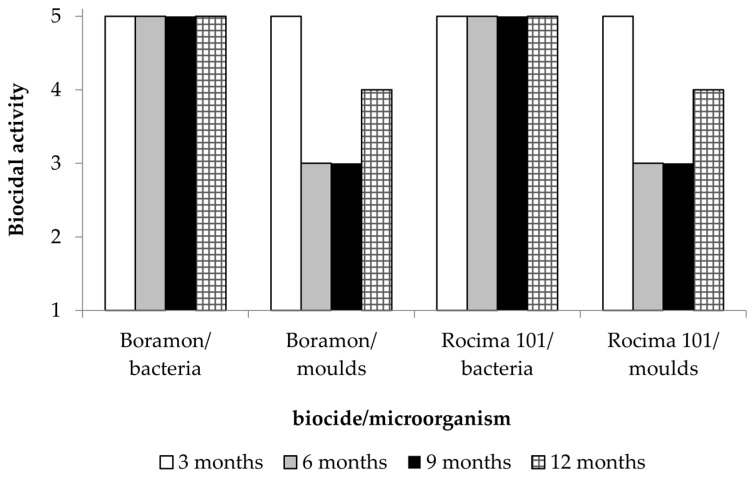
Effectiveness of biocides (Boramon at 30%, Rocima 101 at 8%) applied by spraying.

Under in situ conditions, spraying with 30% Boramon and 8% Rocima 101, effectively protected the historical wood samples against bacteria for up to 12 months (Figure 3). Mould growth on the wood surface was effectively inhibited for a period of three months. After 6 and 9 months, the effectiveness of the disinfectants was estimated at 3 units of biocidal activity, according to the proposed scale (see Section 4.8. Materials and Methods), indicating that mould growth was noted on 50% of the sample surface.

**Figure 4:**
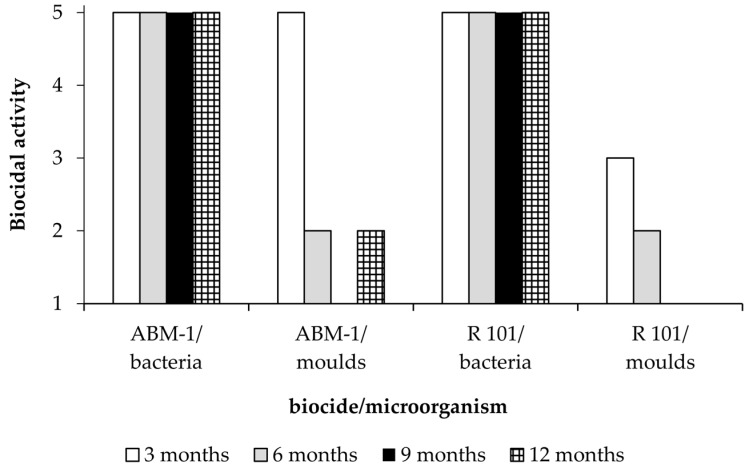
Effectiveness of biocides (ABM-1 at 8%, Rocima 101 at 8%) applied by fogging.

Two disinfectants, Rocima 101 and ABM-1, at a concentration of 8% were applied by fogging. Both of them effectively protected the historical wood samples from bacterial growth for 12 months (Figure 4). The mould growth on the wood surface was suppressed for a period of 3 months after applying ABM-1 at a concentration of 8%. However, more than half of the sample surfaces were covered by moulds as early as three months after application of 8% Rocima 101. 

As with the new material, Boramon was not fogged on the historical material, since its effective concentration of 30% would adversely affect the environment. The methods of application onto wood surfaces were also compared under in situ conditions utilizing Rocima 101. We found that spraying was more effective for protecting against the development of microorganisms on the historical samples.

### 2.6. Evaluation of Changes in the Colour and Luminance of Wood after Application of the Tested Disinfectants

The appropriate disinfectant must not only be selected based on its antimicrobial activity, but the colour and luminance of the treated wood should also be checked after application. Samples of new and historical wood were disinfected by spraying and fogging. Post disinfection treatments, the colour difference (Δ*E*) and change in luminance (Δ*L*) were evaluated by visual and spectrophotometric methods. Values Δ*E* < 2 and Δ*L* < 1 determined by the instrumental method, were considered the levels at which the observer using the visual method does not notice any changes in the colour and luminance of the sample [20].

In the case of the new wood samples disinfected by spraying Boramon and Rocima 101, and by fogging ABM-1 and Rocima 101, there were no statistically significant (*p* < 0.05) colour differences expressed as Δ*E*. The values of Δ*E* < 1 means that the observer did not notice any colour difference. The observed changes in the luminance of the material Δ*L* did not exceed 0.5, which also means that these changes were unnoticeable to the observer (Table 3). The highest colour difference (Δ*E* = 2.82) was observed in the case of the historical material fogged with Rocima 101, where the wood slightly darkened. At the same time, its application by spraying caused no significant change in the colour of the historical wood. 

The statistically significant (*p* < 0.05) differences of luminance and colour between the historical material and the new material after fogging may be connected with the inhomogeneous surface of the historical material. 

## 3. Discussion

Various types of microbiocides are used for wood decontamination and preservation. Modern disinfectants are no longer monocomponent solutions, but mixtures of compounds with multidirectional mechanisms of action. It is unlikely that one synthetic or natural compound will eliminate biological factors adversely affecting wood [13,21]. In the first stage of this study, single compounds (*N*-(3-aminopropyl)-*N*-dodecylpropane-1,3-diamine, didecyldimethylammonium chloride, sodium hypochlorite, hydrogen peroxide, glutaraldehyde, boric acid and lactic acid) were evaluated to determine their activity on microorganisms isolated from the tested wood surfaces. This allowed us to select the compounds with the highest activity. Quaternary ammonium compounds (QACs) inhibited the growth of bacteria and moulds at the lowest concentrations, compared to other substances. The high effectiveness of QACs against moulds, decay fungi and insects that attack wood has been previously described [9,10,15]. These compounds are often used in commercially available wood preservatives. They affect cell membranes, causing the leakage of cell constituents [2,9,22].

In the next phase of the study, Boramon, which additionally contains boric acid, was used in addition to QAC-based preservatives. According to the European Chemicals Agency (ECHA) and the Biocidal Regulation 528/2012, due to its harmful effects on reproduction, this substance is currently used as a biocidal product only for wood preservation (biocides—Category II, Group 8). Boron compounds, however, are often used as wood preservatives [9,10,15,23], and we were able to demonstrate their effectiveness in this study (Figure 1). Boric acid and borates inhibit the function of enzymes and influence cell-to-cell transport mechanisms [2,15].

After evaluating the disinfectant properties, experiments were performed to protect the wood surface against re-infection by bacteria and moulds. In laboratory conditions, preservatives containing QACs: Rocima 101 and ABM-1 at concentrations of 8%, and Boramon at 30%, effectively protected the wood samples against microbial growth despite the high relative humidity of 80% and temperature of 28 °C. However, these results were not reproducible under variable in situ conditions. Similar observations were made by Young et al. [24] who also studied the effect of various biocides on biofilm development on stone substrates, under laboratory and in situ conditions.

In studies on wood preservatives, it is important to check the impact of these compounds on the material. A change in the colour of wood is a measurable parameter and, at the same time, an essential visual element. In the case of new materials, colour differences are readily noticed by consumers and often result in lowering its value. A change in the colour of historical wood caused by the action of chemical compounds may deteriorate its aesthetic value and, above all, contribute to the total destruction of the historical object [2,25,26]. In this study, microbiocides Rocima 101 and Boramon, applied onto the wood samples by spraying, did not change their colour Δ*E* and luminance Δ*L*. The results are lower than those obtained by Tomak et al. [26], who observed the discoloration of pine wood from Δ*E* = 2.29 to Δ*E* = 3.48 under the influence of boric acid at concentrations of 1% and 5%. The authors concluded that the values obtained indicate a slight colour difference. In contrast, Ozgen and Yildiz [27], who used didecyldimethylammonium chloride (DDAC) for pine wood impregnation, reported significant changes in colour and luminance, amounting to 12.2 and 17.8, respectively. In both cases, the wood was subjected to vacuum impregnation.

After removal of the organisms responsible for biodeterioration, it is necessary to provide appropriate environmental conditions. Moisture is one of the main factors contributing to the development of not only mould and bacteria, but also algae and insects. Excess moisture can result from faulty construction of a building, poor site drainage, a leaking roof or a leaking plumbing system, insufficient insulation, or inadequate ventilation. All this may cause rainwater to leak into the interior of the building. Improving the structural condition of the building will significantly reduce the growth of microorganisms on historical materials [21,28,29]. 

**Scheme 1:**
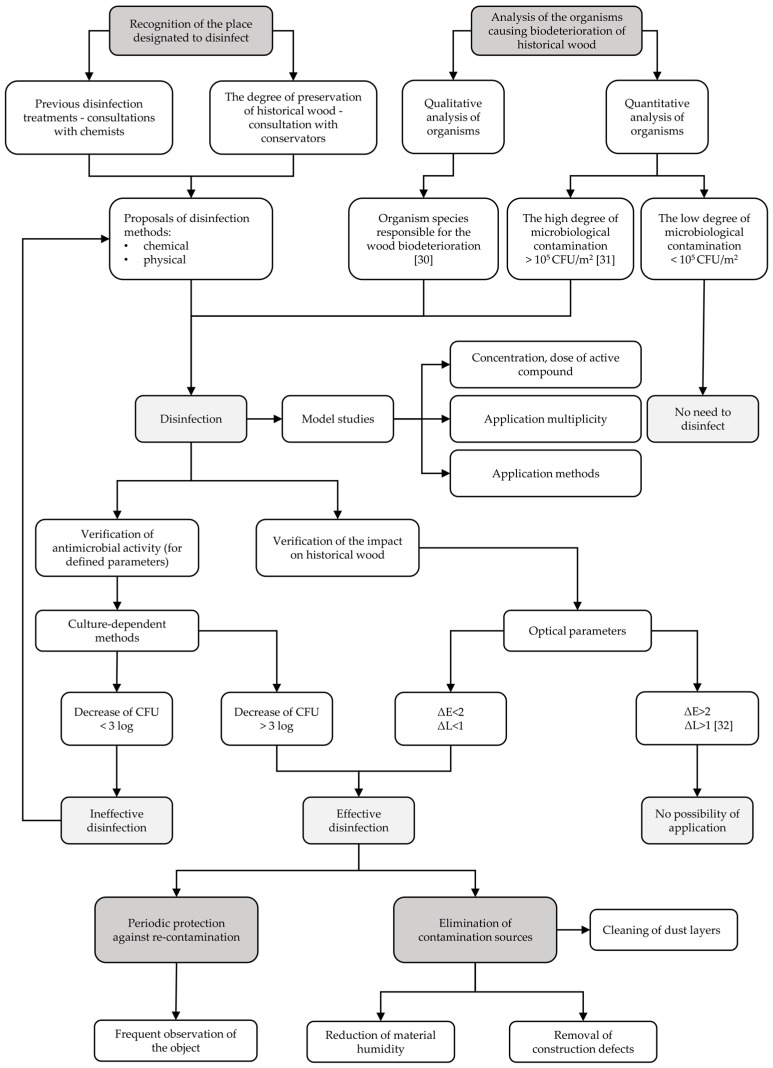
Procedure for the protection of historical wooden buildings from biodeterioration [30,31,32].

In this study, we developed a procedure for the protection of historical wooden buildings from biodeterioration (Scheme 1). Both, biological factors that cause wood biodeterioration and the historical material undergoing deterioration, should first be identified. This will allow the selection of appropriate disinfection methods. When using a variety of chemical compounds, a model study is necessary to determine their concentrations, as well as the number and methods of applications. It is also necessary to check the impact of the biocide on historical material, and its effectiveness after disinfection. Apart from removing the cause of wood degradation, it is crucial to protect the wooden surface against microbial re-infection. Such a comprehensive procedure can contribute to the preservation of many monuments that are invaluable witnesses to history and the past.

## 4. Materials and Methods 

### 4.1. Microorganisms

The effectiveness of active compounds and biocides was tested for three selected strains of bacteria and five strains of moulds, isolated from the wooden surfaces of the historical barracks at the Auschwitz II-Birkenau State Museum in Oświęcim. These include the bacteria *Pseudomonas fluorescens*, *Staphylococcus equorum*, and *Bacillus cereus*; and moulds *Alternaria alternata*, *Chaetomium globosum*, *Cladosporium cladosporioides*, *Engyodontium album*, and *Penicillium citreonigrum*. The nucleotide sequences of the 16S rRNA gene of the bacteria used in the study were deposited in GenBank, the National Centre for Biotechnology Information (*Pseudomonas fluorescens* KM036083.1; *Staphylococcus equorum* KM036089.1; *Bacillus cereus* KM036070.1). Mould strains were deposited in the Culture Collection ŁOCK 105 under the collection numbers *Alternaria alternata* ŁOCK 0594, *Chaetomium globosum* ŁOCK 0591, *Cladosporium cladosporioides* ŁOCK 0592, *Engyodontium album* ŁOCK 0590, *Penicillium citreonigrum* ŁOCK 0597. The bacteria were maintained on tryptic soy agar slants (TSA, Merck, Germany) and the moulds were stored on malt extract agar slants (MEA, Merck, Germany) at 4 °C. In order to activate the strains, the biomass was collected from slants and sub-cultured: the bacteria into tryptic soy broth (TSB, Merck, Germany) and the moulds onto MEA slants. The cultures were incubated at 30 °C for 24–48 h (bacteria), and 28 °C for 5 days (moulds).

### 4.2. Determining the Minimum Inhibitory Concentrations of Active Compounds in Biocides

Antimicrobial activity was determined for seven commercially available active compounds, selected on the basis of biocides listed on the website of The Office for Registration of Medicinal Products, Medical Devices and Biocidal Products (http://bip.urpl.gov.pl/pl/biuletyny-i-wykazy/produkty-biob%C3%B3jcze). These included didecyldimethylammonium chloride (CAS 7173-51-5, LONZA AG, Basel, Switzerland), *N*-(3-aminopropyl)-*N*-dodecylpropane-1,3-diamine (CAS 2372-82-9, LONZA AG, Basel ,Switzerland), hydrogen peroxide (CAS 7722-84-1, Evonik, Essen, Germany), glutaraldehyde (CAS 111-30-8, BASF, Ludwigshafen, Germany), sodium hypochlorite (CAS 7681-52-9, Solvay SA, Brussels, Belgium), boric acid (CAS 10043-35-3, Alfa Aesar, Karlsruhe, Germany) and l-lactic acid (CAS 79-33-4, Sigma Aldrich, St. Louis, MO, USA). The concentrations of these active compounds were determined based on the concentrations of these ingredients in various commercially available biocides in Poland (Table 4).

The sensitivity of microorganisms to the active compounds was determined by the disc-diffusion assay. The surfaces of TSA and MEA were inoculated with bacterial and mould monocultures, respectively (10^6^ CFU/mL and 10^6^ conidia/mL), which were then uniformly spread on the surfaces of the media. Sterile paper disks (ø 6 mm, Oxoid) were soaked with 15 mL solutions of the compounds, at the test concentrations (Table 1); subsequently, the discs were placed on the surface of the media. The plates were incubated at 30 °C for 24–48 h (bacteria) and at 28 °C for 48 h (moulds). Macroscopic observations of microbial growth were carried out, and the diameters of inhibition zones were measured. The MIC value was the lowest tested concentration of an active ingredient, for which the inhibition zone was observed with a diameter ≥ 10 mm.

### 4.3. Determining the Antimicrobial Activity of Biocides by Disc Diffusion Method

Eight commercial biocides, containing didecyldimethylammonium chloride as the active compound and various excipients, were used in the study (Table 5). Each biocide was tested at concentrations recommended by its manufacturer.

Antimicrobial activity of biocides was tested by the disc diffusion method, as described in Section 4.2. Mixed populations of bacteria and mixed populations of moulds were tested. The bacterial cultures activated on TSB were centrifuged (6000× *g*, 10 min), and the biomass was suspended in saline solutions (0.85% NaCl). Bacterial strains were combined in equal volumes to obtain mixed cultures. The density of the suspension was adjusted to 10^8^ CFU/mL. The MEA slants of five-day fungal cultures were washed with sterile distilled water supplemented with 0.01% of Tween80. The density of the mould inoculum in the mixed cultures was determined using a haemocytometer, and adjusted to 10^6^ conidia/mL.

### 4.4. New and Historical Wood

Samples of new material, in the form of white poplar wood fragments measuring 50 × 20 × 10 mm, were used in the study. The samples were sterilized twice at 121 °C for 15 min., and then stabilized in a constant climate chamber (Binder, Germany) for seven days, at 28 °C and relative humidity (RH) 80%. However, historical wood fragments, collected from the Auschwitz II-Birkenau State Museum in Oświęcim, were used under in situ conditions.

### 4.5. Activity of Biocides against Microorganisms Colonizing Wood Surface

The mixed cultures (1 mm each) of bacteria (10^8^ CFU/mL) and moulds (10^6^ conidia/mL), activated in the media ((NH_4_)_2_SO_4_ 0.075%, K_2_HPO_4_ 0.025%, MgSO_4_·7H_2_O 0.125%, yeast extract 0.125%, glucose 0.5% and agar 0.1% pH 6.0), were applied onto the surface of new wood samples [22]. The samples were incubated in a constant climate chamber with a relative humidity of 80% and a temperature of 28 °C for 7 days (bacteria) and 21 days (moulds). After incubation, each of the test biocides at two concentrations, was sprayed (using a professional sprayer Mercury Super Pro 360, Quasar, Poland) onto the wood surface one, two, and three times, at intervals of 24 h. The number of microorganisms was determined after successive applications of each biocide, by the contact plate method in TSA (bacteria) and MEA (moulds). The cultures were incubated at 30 °C for 48 h (bacteria) and at 28 °C for 5 days (moulds). Control samples were materials not subjected to the biocide treatment.

### 4.6. Protecting New Wood Samples against Microorganism Growth: Laboratory Tests

Selected biocides (ABM-1 8%, Boramon 30%, Rocima 101 8%) were applied by the spraying method to the surface of each sterile, conditioned sample of new wood (two times at an interval of 30 min) or fogging method (in accordance with the procedure: 1 g of biocide per 1 m^3^ of air with the addition of 5% of MIST-60 containing polyols). Twenty-four hours after the application of biocides, the sample surfaces were inoculated with mixed cultures of bacteria (10^8^ CFU/mL) and moulds (10^6^ conidia/mL). The samples were incubated in a constant climate chamber at 28 °C and 80% RH for seven days, one month, three months and six months. The effectiveness of antimicrobial activity was determined by the contact plate method, as described above. Control samples were materials not subjected to the biocide treatment.

### 4.7. Protecting Historical Wood Samples against Microorganism Growth—In Situ Tests

Selected biocides (ABM-1 8%, Boramon 30%, Rocima 101 8%) were applied onto the surface of each sterile, conditioned sample of historical wood by the spraying method and by the fogging method. The samples were placed on metal shelves in the washroom located in the western part of the brick barracks B-65 at the Auschwitz II-Birkenau State Museum (Figure 1). The effectiveness of these biocides against the growth of microorganisms present in the museum environment was determined by the contact plate method after 3, 6, 9 and 12 months of storage under in situ conditions. Control samples were historical wood not subjected to the biocide treatment.

### 4.8. The Evaluation Scale for the Antimicrobial Activity of the Biocides Applied to the New and Historical Wood Surfaces

In order to determine the activity of the biocides against bacteria and moulds, a calibration scale was used according to Table 6.

### 4.9. The Impact of Biocides on the Wood Colour

The change in colour and luminance of wood samples was quantified by the spectrophotometric method [32,33]. The theoretical colour model was developed by the International Commission on Illumination (CIE). The model takes into account all the colours recognizable by the human eye, including all RBG and CMYK colours. The CIE model is a three-dimensional colour space, which is described by three parameters:

*L*—luminance ranging from 0 (black) to 100 (white).
*a*—the scope from red to green.
*b*—the scope from yellow to blue.

The Δ*E_00_* model was used to describe colour differences. In addition to the trichromatic components, it takes into account four characteristics: saturation, hue, brightness and blue colour. The following correlation was used to develop the results. It is the Euclidean distance between two points in a three-dimensional space of colour:

$$\Delta E=\sqrt{{\left(\Delta L\right)}^{2}+{\left(\Delta a\right)}^{2}+\;{\left(\Delta b\right)}^{2}}$$

Trichromatic components were measured at three points of each tested area, and the results were averaged. Samples were analysed by a spectrophotometer (Konica Minolta CM-2500d).

Measurements were performed on the new and historical wood samples before biocide application, after two applications of Boramon and Rocima 101 by the spraying method, and after one application of ABM 1 and Rocima 101 by the fogging method. Control samples consisted of historical wood not subjected to the biocide treatment. 

The results of Δ*E* and Δ*L* represent means from three independent samples ± SD. Differences between means were tested by variance analysis (one-way ANOVA) with the post-hoc Tukey test. Probability (*p*) values of <0.05 were considered significant. Statistica v.10.0 (Stat Soft. Inc., Tulsa, OK, USA) was used for calculations.

## 5. Conclusions

In this work, effective and safe microbiocides for the disinfection and protection of historical wooden surfaces at the former Auschwitz II-Birkenau concentration and extermination camp were indicated. Their concentration, times and method of application has been taken into account and the impact of microbiocides on historical material was examined. The most effective were didecyldimethylammonium chloride and *N*-(3-aminopropyl)-*N*-dodecylpropane-1,3-diamine. The majority of the microbiocides (six of eight tested) contain these compounds and in laboratory conditions they effectively inhibit the growth of microorganisms after triple application by spraying. In addition, in situ Boramon 30% and Rocima 101 8% applied by spraying, effectively protected the historical wood from growth of bacteria for 12 months and moulds for 3 months. In the article, a monitoring scheme for the protection of historical wooden buildings from biodeterioration was suggested. It covers all stages of comprehensive recognition of the same object from disinfection to conservation. The presented procedure may help to perform similar analyses by other researchers.

## Figures and Tables

**Table 1:** Minimal inhibitory concentration of active compounds in % (*v*/*v*).

Microorganism	Compound						
	DDAC	APDA	HP	GA	SH	BA	LA
*Bacillus cereus* KM036070.1	0.02	0.02	2	2	0.2	5	4
*Pseudomonas fluorescens* KM036083.1	7	0.02	2	2	0.2	2	4
*Staphylococcus equorum* KM036089.1	0.02	0.02	2	2	0.2	8	4
*Alternaria alternate* ŁOCK 0594	0.2	0.2	2	2	2	2	>10
*Cladosporium cladosporioides* ŁOCK 0592	0.2	0.2	2	4	0.2	12	>10
*Engyodontium album* ŁOCK 0590	0.2	0.2	0.2	0.2	0.02	8	10
*Penicillium citreonigrum* ŁOCK 0597	2	2	2	0.2	2	2	>10
*Cheatomium globosum* ŁOCK 0591	0.2	0.2	0.2	2	7	2	>10

DDAC—didecyldimethylammonium chloride, APDA—*N*-(3-aminopropyl)-*N*-dodecylpropane-1,3-diamine, HP—hydrogen peroxide, GA—glutaraldehyde, SH—sodium hypochlorite, BA—boric acid, LA—lactic acid.

**Table 2:** Concentrations of biocides (%, *v*/*v*) where growth inhibition zone ≥ 10 mm in diameter was observed.

Microorganisms	Biocide							
	ABM-1	ABM-2	A LV 706	AT	B	M	P R80	R101
Bacteria	1	1	10	1	15	10	2.5	1
Moulds	6	6	10	6	20	20	6	6

ALV706—Acticide LV 706; AT—Atoxyn; B—Boramon; M—Mycetox B’; P R80—Preventol R80; R101—Rocima 101.

**Table 3:** The colour and luminance of wood after application of disinfectants.

Application	Biocide	New Material		Historical Material	
		Δ*E*	Δ*L*	Δ*E*	Δ*L*
Spraying	Boramon	0.44 ± 0.31 ^a^^,^^A^	−0.43 ± 0.19 ^a,B^	0.23 ± 0.06 ^a,A^	−0.07 ± 0.02 ^a,C^
	Rocima 101	0.45 ± 0.21 ^a,A^	−0.42 ± 0.28 ^a,b,B^	0.78 ± 0.33 ^b,A^	−0.63 ± 0.13 ^b,C^
Fogging	ABM-1	0.49 ± 0.29 ^a,A^	−0.15 ± 0.02 ^b,C^	1.38 ± 0.34 ^b,B^	−1.38 ± 0.25 ^c,D^
	Rocima 101	0.48 ± 0.23 ^a,A^	−0.33 ± 0.17 ^a,b,C^	2.82 ± 0.99 ^c,B^	−1.81 ± 0.53 ^c,D^

Trichromatic components: Δ*E*—colour difference; Δ*L*—luminance difference; values in the table represent means from three samples ± SD; lowercase letters (^a–c^) in the columns indicate statistically significant differences in the Δ*E* or Δ*L* means within disinfectants and application methods (one-way Anova, *p* < 0.05); capital letters (^A–D^) in the rows indicate statistically significant differences in the Δ*E* or Δ*L* means within various types of material (one-way Anova, *p* < 0.05).

**Table 4:** Active chemical compound of biocides.

Compound	Acronym	Concentration Used in the Tests (% *v*/*v*)
Didecyldimethylammonium Chloride	DDAC	12, 10, 7, 3, 2, 0.2, 0.02
*N*-(3-aminopropyl)-*N*-dodecylpropane-1,3-diamine	APDA	5, 2, 0.5, 0.2, 0.02
Hydrogen Peroxide	HP	15, 10, 7, 5, 2, 0.2, 0.02
Glutaraldehyde	GA	10, 5, 4, 2, 0.2, 0.02
Sodium Hypochlorite	SH	7, 5, 2, 0.2, 0.02
Boric Acid	BA	12, 8, 5, 2, 0.2, 0.02
l-lactic Acid	LA	10, 8, 4, 2, 0.2

**Table 5:** Biocides used in the tests.

Biocide	Active Components	Concentrations (% *v*/*v*)
ABM-1 (MDA Sp. z o.o., Poland)	*N*-3-aminopropyl-*N*-alkyl(C10-C14)-1,3-propanediamine *N*,*N*-dialkyl(C10-C16)-*N*-methyl-*N*-poly(oxyethylene)ammonium propionate *N*,*N*-dialkyl(C10-C14)-*N*,*N*-dimethyl ammonium chloride 2-[3-(dodecanoylamino)]propyl dimethyl ammonium acetate	1, 2, 4, 6, 8
ABM-2 (MDA Sp. z o.o., Poland)	*N*-3-aminopropyl-*N*-alkyl(C10-C14)-1,3-propanediamine *N*,*N*-dialkyl(C10-C16)-*N*-methyl-*N*-poly(oxyethylene)ammonium propionate *N*,*N*-dialkyl(C10-C14)-*N*,*N*-dimethyl ammonium chloride	1, 2, 4, 6, 8
Acticide LV 706 (THOR GmbH, Germany)	2.5%–10% (*v*/*v*) (benzyl alkyl(C_8–18_) dimethylammonium chlorides 2.5%–10% (*v*/*v*) diethylene glycol <2.5% (*v*/*v*) 2-octyl-2*H*-isothiazole-3-one	10, 15, 20
Atoxyn (Polfa S.A., Poland)	10% (*v*/*v*) benzyl alkyl (C8–18)dimethylammonium bromides	1, 2.5, 6, 8, 12
Boramon (Altax Sp. z o.o., Poland)	24% (*v*/*v*) benzyl alkyl(C12–16)dimethylammonium chlorides 5% (*v*/*v*) boric acid	10, 15, 20, 30
Mycetox B’ (ADW Sp. z o.o., Poland)	<9.5% (*v*/*v*) *N*,*N*-didecyl-*N*,*N*-dimethylammonium chloride 2% (*v*/*v*) citric acid; 0.5% (*v*/*v*) (2-metoksymetyloetoksy)propanol	10, 20, 30
Preventol R80 (Bayer AG, Germany)	80% (*v*/*v*) benzyl alkyl dimethylammonium chloride 8%–12% (*v*/*v*) (2-methoxymethylethoxy)propanol	2.5, 6, 12
Rocima 101 (H.S.H. Sp. z o.o., Poland)	40%–<60% (*v*/*v*) *N*,*N*-didecyl-*N*,*N*-dimethylammonium chloride 20%–<25% (*v*/*v*) isopropanol	0.5, 1, 2, 6, 8

**Table 6:** The method of evaluating antimicrobial activity of biocides.

Biocidal Activity	Bacteria	Reduction in the Number of Bacteria	Moulds	% of the Surface Contamination of Samples
High 5	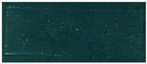	8 log	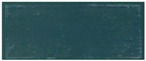	no growth
Good 4	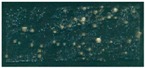	6 log	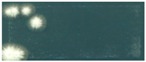	25%
Low 3	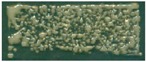	4 log	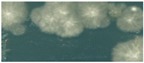	50%
Very Low 2	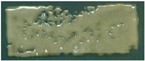	2 log	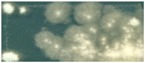	75%
No Activity 1	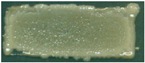	no reduction	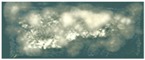	100%
